# Carotid artery calcific disease management strategies during transcarotid artery revascularization

**DOI:** 10.1590/1677-5449.210152

**Published:** 2021-03-12

**Authors:** Rafael Demarchi Malgor, Nicolas J. Mouawad

**Affiliations:** 1 University of Colorado, Division of Vascular Surgery and Endovascular Therapy, Aurora, CO, United States of America.; 2 McLaren Health System, Division of Vascular & Endovascular Surgery, Bay Region, Bay City, MI, United States of America.; 3 Michigan State University, Division of Vascular Surgery, East Lansing, MI, United States of America.

To the Editors,

Endovascular treatment of carotid artery disease has gone through fast-paced evolution since the advent of balloon angioplasty and stents three decades ago. Nevertheless, it was not until significant improvements in neuroprotection were achieved that carotid artery stenting became a solid contender to the gold standard carotid artery endarterectomy. Embolic protection devices, such as proximal common and/or external carotid artery balloon occlusion devices and internal carotid artery filters paved the way toward a safer and more definitive strategy. The concept of flow reversal in the early 2000s,^1^ initially conceptualized by Juan Parodi, has been refined and has culminated contemporarily in the development of a new flow reversal transcarotid artery revascularization and stenting system.^2^

Carotid artery calcific disease remains a challenge in patients undergoing carotid artery stenting regardless of whether it is done via a transfemoral or transcarotid approach. By far, the most challenging scenario is circumferentially calcified internal carotid artery plaques that can cause inadequate expansion of stents and limited luminal gain despite vigorous pre- and post-dilation of the lesion.^3^ Significant residual stenosis (>30%) and incomplete expansion of the stent can largely compromise the durability of the intervention.^4^ Off-label use of FDA-approved, commercially available devices for treatment of calcific lower extremity peripheral arterial disease can be successfully utilized to facilitate carotid artery stenting when compassionate use can be justified.

The two main strategies use either atherectomy devices or the new intravascular lithotripsy (IVL) balloon. We have reported both use of orbital atherectomy^5^ and IVL balloon angioplasty^6^ during transcarotid artery revascularization (TCAR) in patients with severe carotid artery calcific disease with success. In patients not suitable for open revascularization, our algorithm is to consider use of orbital atherectomy in patients with heavily calcified, eccentric plaques, reserving the IVL technology to treat patients with circumferentially calcified carotid artery lesions. We recommend that flow reversal be maintained a minute longer than usual during those cases, while keeping systolic blood pressure between 140 and 160mmHg to create a better arterial-venous gradient in order to ensure removal of a larger amount of calcium-based debris. A further step toward large-scale trials employing both technologies to treat carotid artery calcific disease is warranted.
